# Supplementary material for: Polish Adaptation of the Italian Spine Youth Quality of Life Questionnaire
Source: J Clin Med. 2021 May 12;10(10):2081. doi: 10.3390/jcm10102081 (PMC8151829; doi:10.3390/jcm10102081)
Supplement: Supplementary file 1 [file jcm-10-02081-s001.zip › jcm-1205651-supplementary.pdf]

Kwestionariusz ISYQOL wersja polska

Imię_______________ Nazwisko_______________ ____________ Data_______________

Chcielibyśmy ocenić twoje samopoczucie biorąc pod uwagę stan zdrowia twojego kręgosłupa. Spróbuj samodzielnie odpowiedzieć na następujące pytania:

1. Czy obawiasz się, że twój problem z kręgosłupem może się pogorszyć ?

□ NIGDY □ CZASAMI □ CZĘSTO

---

2. Czy obawiasz się bólu kręgosłupa w życiu dorosłym z powodu twojego problemu ?

□ NIGDY □ CZASAMI □ CZĘSTO

---

3. Czy uważasz, że twój problem z kręgosłupem to masakra / koszmar (coś bardzo poważnego) ?

□ NIGDY □ CZASAMI □ CZĘSTO

---

4. Czy martwisz się, że pomimo twoich starań twój kręgosłup nie będzie zdrowy ?

□ NIGDY □ CZASAMI □ CZĘSTO

---

5. Czy uważasz, że istnieją poważniejsze problemy zdrowotne niż twój z kręgosłupem ?

□ NIGDY □ CZASAMI □ CZĘSTO

---

6. Czy pomimo problemu z kręgosłupem prowadzisz normalne życie ?

□ NIGDY □ CZASAMI □ CZĘSTO

---

7. Czy obecnie odczuwasz ból kręgosłupa ?

□ NIGDY □ CZASAMI □ CZĘSTO

---

8. Czy wygląd twoich pleców sprawia, że czujesz się niekomfortowo ?

□ NIGDY □ CZASAMI □ CZĘSTO

---

9. Czy martwisz się stanem zdrowia twojego kręgosłupa ?

□ NIGDY □ CZASAMI □ CZĘSTO

---

10. Czy zdarza ci się myśleć, że problem twojego kręgosłupa nie jest aż tak poważny ?

□ NIGDY □ CZASAMI □ CZĘSTO

---

11. Czy wstydzisz się pokazywać twoje ciało ?

□ NIGDY □ CZASAMI □ CZĘSTO

---

12. Czy martwisz się, że problem twoich pleców jest bardzo widoczny ?

□ NIGDY □ CZASAMI □ CZĘSTO

---

13. Czy pomimo problemu z kręgosłupem prowadzisz szczęśliwe życie ?

□ NIGDY □ CZASAMI □ CZĘSTO

Jeżeli nie nosisz gorsetu kwestionariusz kończy się tutaj. Jeżeli nosisz gorset z powodu problemów z plecami, odpowiedz na następujące pytania:

14. Czy z powodu gorsetu nie możesz się ubierać tak jak chcesz ?

□ NIGDY □ CZASAMI □ CZĘSTO

---

15. Czy martwisz się, że gorset może być widoczny pod ubraniem ?

□ NIGDY □ CZASAMI □ CZĘSTO

---

16. Czy czujesz się przygnębiony/a ponieważ od kiedy nosisz gorset nie robisz rzeczy, które robiłeś/aś wcześniej ?

□ NIGDY □ CZASAMI □ CZĘSTO

---

17. Czy gorset ogranicza twoje ruchy ?

□ NIGDY □ CZASAMI □ CZĘSTO

---

18. Czy zdarza ci się płakać z powodu gorsetu ?

□ NIGDY □ CZASAMI □ CZĘSTO

---

19. Czy czujesz się nieakceptowany/a przez innych z powodu noszenia gorsetu ?

□ NIGDY □ CZASAMI □ CZĘSTO

---

20. Czy noszenie gorsetu jest niewygodne ?

□ NIGDY □ CZASAMI □ CZĘSTO

Jeżeli chcesz, wykorzystaj to miejsce na dodatkowy komentarz:
